# Support Needs of Mothers of Children with Down Syndrome in Kuwait

**DOI:** 10.3390/healthcare14203368

**Published:** 2026-10-09

**Authors:** Sana A. AlBustan, Hadeel S. Ayyad, Fatima S. Afaar, Ghezlan A. Alostath, Dana K. AlGhareeb, Hussain A. Abdulla

**Affiliations:** 1 Department of Communication Disorders Sciences, Faculty of Life Sciences, Kuwait University, Sabah AlSalem University City, Shadadiya, P.O. Box 5069, Safat 13060, Kuwait; 2 Faculty of Medicine and Health, The University of Sydney, Science Rd, Camperdown NSW 2050, Australia; 3 Alfekrya School for Special Needs, Ministry of Education, Hawalli 900002, Kuwait; 4 Independent Researcher, Manama, Bahrain

**Keywords:** Down syndrome, mothers, primary caregiver, family support, speech-language pathology, Family Needs Questionnaire (FNQ), Kuwait

## Abstract

**Highlights:**

**What are the main findings?**

- Mothers of children with Down syndrome reported high support needs across all domains.
- Family size is associated with the social needs of mothers with a child with Down syndrome, which reflects the effect of siblings in providing support and sharing caregiving responsibilities.

**What are the implications of the main findings?**

- Caregiver demands following a Down syndrome diagnosis should be viewed as multidimensional.
- Coordinated family-centered services are needed to address caregiver needs.

**Abstract:**

**Background objective:** This study explored the level of support needs of mothers of children with Down syndrome (DS) in Kuwait following postnatal diagnosis, across four domains: educational needs, professional needs, social needs, and primary caregiver support needs. **Methods:** A cross-sectional survey was conducted with 82 mothers of children with DS residing in Kuwait. Descriptive statistics were calculated, while group comparisons (in terms of needs) were conducted using nonparametric tests, and internal consistency was assessed using Cronbach’s alpha. Reliability estimates ranged from α = 0.79 to α = 0.89 across the four subscales, with an overall alpha of 0.93. **Results:** Participants reported high levels of need across all domains, including educational, professional, social, and primary caregiver support needs, with a median score of 5 on a 1–5 scale (Likert). The most prominent finding in group comparison was the significant association between family size and the social needs score (*p* = 0.012), where the median was 5 (IQR: 4.89–5.00) for family size < 3, and it decreased as we moved towards larger families, until it reached 3.78 for family size > 8. **Conclusions:** Caregiver demands following a postnatal DS diagnosis were multidimensional and strongly interconnected. Findings underscore the importance of coordinated post-diagnosis services that addressed informational, professional, social, and practical support needs for mothers and primary caregivers.

## 1. Introduction

### 1.1. Background

The Centre for Arab Genomic Studies (2013) [1] reported that the incidence of Down syndrome (DS) in several Arab countries is higher than international estimates, with the incidence in Kuwait estimated at approximately 1 in 581 live births. Corder et al. (2017) [2] similarly reported incidence rates ranging from 1 in 449 to 1 in 554 across various GCC countries, excluding Bahrain. In comparison, a recent CDC report [3] estimated the US rate at approximately 1 in 621 live births. These incidence rates indicate that Down syndrome is a relatively common congenital condition in the region and that families affected by DS are likely to be found across different communities and districts. Parents in these families may face particular circumstances and challenges associated with raising a child with DS.

Parenting, often described as a rewarding yet overwhelming experience, is accompanied by fluctuating levels of strain and challenges owing to the day-to-day demands of being a parent [4]. The birth of a child with disability brings about unexpected demands, responsibilities, and challenges to parents, many of whom may not be prepared. Having a child with disabilities brings about changes to the lives of the entire family [5,6,7].

### 1.2. Multidimensionality of Parents’ Needs and Aim of the Study

Existing research has shown that parents of children with DS frequently report unmet needs in the period following diagnosis. These needs are not confined to medical information alone, but often include emotional reassurance, access to full professional knowledge, practical caregiving support, and opportunities for social and community connection. A validation study further supports this multidimensional view of family needs, showing that families of infants with disabilities may have needs related to information, social support, community services, financial assistance, explaining the disability to others, and family functioning [8]. Taken together, these findings suggest that parental needs following the diagnosis of DS are multidimensional and encompass educational, professional, social, and practical caregiving aspects.

Accordingly, this study explored the level of support needs of mothers of children with DS in Kuwait following postnatal diagnosis, across four domains: educational needs, professional needs, social needs, and primary caregiver support needs.

### 1.3. Educational Needs

Knowledge and training constitute an important aspect of parents’ role in relation to the development of their child with Down syndrome and their ability to make appropriate decisions that serve the child’s best interests. Accordingly, many authors such as Hart and Neil (2003) have given considerable attention to knowledge as an important dimension of parents’ needs [9].

It should be noted that the need for obtaining the right information starts from the time of diagnosis (and not post-diagnosis only). Many healthcare providers still tend to emphasize the limitations of intellectual disability when communicating the diagnosis of DS [10]. In the Kuwaiti context, while the information provided to parents may be partially accurate in many cases, it is not necessarily reviewed by medical experts nor endorsed by any national or local DS organization. Consequently, this can lead parent(s) to make decisions based on incomplete and/or uninformed information. For these reasons, it is critical that healthcare providers offer accurate, expert-reviewed information to anyone receiving a DS diagnosis. Another study on the preliminary reactions of parents to their child’s diagnosis of DS found that the parents’ reactions to the diagnosis involved a range of intense preliminary emotions, including grief, denial, guilt, and anger [11]. Most parents had negative experiences with medical professionals and argued that they did not receive sufficient support from medical professionals. They reported a lack of accurate and updated information about DS, whereby information about DS was actively sought by the parents. These reported shortcomings in information and support also point to the importance of health professionals’ preparation in communicating a DS diagnosis. Studies involving obstetricians, pediatricians, residents, and midwives have shown that many clinicians feel unprepared and uncomfortable when delivering a DS diagnosis, which may result in inconsistent and sometimes insensitive disclosure practices. Research conducted in Turkey demonstrated that pediatric residents from an Eastern country felt uncomfortable when delivering information on the diagnosis of newborns with DS to their families [12]. The study further found that female residents felt a higher sense of discomfort; however, they were more willing to receive training in delivering difficult news.

### 1.4. Professional Needs

One of the most evident dimensions of the needs of parents of a child with Down syndrome is the need for experienced and specialized professionals, as knowledge alone is not sufficient without guidance and the presence of an advisor throughout the parents’ journey with their child. For this reason, professional support has been considered a main domain of parental needs in several Family Needs Questionnaires (FNQs), such as those used by Wolf (2009) [13] and Lee et al. (2021) [14].

These professional needs extend beyond the initial diagnosis, as caregivers must continue to seek reliable information, navigate services, manage the demands of care, and adapt family life to meet the developmental needs of their child with DS. More recently, it has been emphasized that families benefit from accurate information, guidance, and support following diagnosis [15].

Evidence from the Gulf region suggests that continuity of such support may be limited after the initial diagnosis. AlShatti et al. (2021) [16] examined the experiences of caregivers of individuals with Down syndrome in Kuwait; Alsubaihi et al. (2024) [17] discussed the broader healthcare context across the Gulf region, with greater emphasis on Saudi Arabia; and Al-Kuwari et al. (2021) [18] examined the situation in Qatar. Collectively, these studies identified challenges including communication and information-exchange problems between healthcare providers, difficulties accessing specialized services, limited availability of specialized services in some areas, inadequate guidance following diagnosis, prolonged waiting times for investigations and specialist appointments, and difficulties experienced by caregivers in managing their children’s health problems. These findings suggest that families may face considerable challenges in accessing and navigating the specialized and continuing care required by children with Down syndrome.

These gaps highlight the importance of healthcare professionals as a continuing source of guidance and as a link to appropriate services. Pediatricians who communicate diagnoses comprehensively and connect families to health and community care services may develop stronger therapeutic relationships with patients and their families, positioning them as a critical first line of post-diagnostic support [15,19].

Professional support may also be needed when families begin to identify and access services for the child’s emerging developmental needs. Children with DS frequently experience difficulties in speech, language, and communication development, particularly in expressive language and broader communication skills, which may affect later learning and social participation [20]. These difficulties have been linked to anatomical differences, oral motor difficulties, hearing problems, and short-term memory limitations [21]. Since such developmental needs may emerge early, families may require timely guidance regarding referral to appropriate communication services. Speech-language pathology and speech-language therapy may therefore form part of the professional support needed by primary caregivers following diagnosis [22]. Clear professional guidance may help families understand available services, access appropriate care, and remain engaged with developmental services as their child’s needs evolve [15].

### 1.5. Social Needs

Additionally, many authors have pointed out that a limited social environment, in terms of the number of people and children with whom a child interacts, may hinder the development of a child with Down syndrome [23]. Like any other child, children with DS need a sense of belonging and love, through intimate relationships, friendships, and other forms of social connection, which are among the needs situated in the middle of Maslow’s hierarchy of human needs. The issue ultimately depends on the availability of an environment that understands these children and knows how to interact with, support, and include them—insights that can be drawn from the study by AlShatti et al. (2021) [16].

Such social connection and inclusion are important not only for the child’s development, but also for supporting the child’s emotional well-being and sense of belonging. Children with DS, like all children, have individual strengths, emotions, and interests, and can benefit from opportunities that support their development and well-being based on the level of their unique disability. They have the same emotions and interests in learning, playing, and enjoying a strong sense of well-being and belonging [24].

The social environment surrounding the child may also affect the experiences of parents, who may face additional stress when managing the demands associated with raising a child with a disability. Raising a child with a disability can place additional stress on parents [25,26]. Social support can help protect parents from the negative effects of these challenges [25,26]. In addition, the ability of families to cope with these difficulties may influence parents’ participation in intervention programs [27].

Accordingly, support within the family and broader social environment may be particularly important in helping parents manage these demands. Parental stress has been found to be closely related to the level of support available within the family [28]. Similarly, parents of children with developmental disabilities may experience strain in family functioning and depend on social support to manage these demands [29].

Hence, these findings suggest that social needs encompass both the child’s opportunities for connection, inclusion, and belonging and the parents’ access to supportive relationships and social resources that help them manage the demands of caregiving.

### 1.6. Caregiver Support Needs

Researchers have also recognized the parents’ need for rest and relief from the burden of caregiving, given the additional and intensive care they provide for their child with Down syndrome—an issue highlighted by Bodde et al. (2025) [30]. This includes the need for adequate sleep, reduced working hours, leave from work, and assistance from others in caring for the child, such as a spouse, other family members, a domestic helper, grandparents, or others. Wolf (2009) [13] identified a distinct “Personal Domain” that included needs related to parents’ personal time and respite from caregiving.

### 1.7. Rationale of the Study

While a number of studies regarding the support needs of caregivers of children with DS are quite large globally, it is under-explored in the Gulf region, including Kuwait. At the time this study was designed and data collection initiated in 2020, research examining post-diagnosis support needs of mothers and primary caregivers of children with Down syndrome in the Gulf region was extremely limited. Based on our search of the literature, we identified that no directly relevant studies were published in the Gulf region prior to 2020 that specifically examined caregivers’ support needs following a diagnosis of Down syndrome. Since 2020, a small number of studies addressing this topic have emerged, including AlShatti et al. (2021) [16] in Kuwait and Alabri (2023) [31] in Saudi Arabia; however, both studies adopted qualitative approaches. Other studies published during this period have addressed related but distinct issues, including disability more generally [32], quality of life [33], and caregiving costs [34], rather than caregivers’ support needs as their primary focus. Thus, although a small body of directly relevant research has emerged since 2020, the evidence remains limited, particularly quantitative evidence examining post-diagnosis support needs across multiple domains. The present study, therefore, contributes quantitative evidence on mothers’ and primary caregivers’ support needs following a postnatal diagnosis of Down syndrome, examining these needs across four domains: educational, professional, social, and primary caregiver support.

## 2. Materials and Methods

This research used a cross-sectional survey design to examine perceived support needs among mothers of children with DS in Kuwait following postnatal diagnosis, with the final sample primarily reflecting maternal perspectives. Eligible participants were adults aged 18 years or older who were the parents or primary caregivers of a child with DS residing in Kuwait and whose child had received postnatal diagnosis.

To collect data for the present study, convenience sampling was used to recruit 86 parents of children with DS from various locations in Kuwait over a three-month period. Names and contacts of participants were taken from the Down Syndrome Society in Kuwait, which maintains records of parents of children with DS from all six governorates in Kuwait. As the vast majority of respondents were mothers (*n* = 82, 95.35%), the four fathers were excluded from the analysis to maintain a consistent focus on mothers and facilitate a clear interpretation of the findings. Thus, the final sample comprised 82 mothers of children with DS, of whom the majority were married (85.37%) with household sizes of 3–5 members (65.85%). Surveys that were incomplete or inconsistent in responses were excluded from the sample size prior to any running of statistical data.

Although no hypotheses were specified a priori, a test-based post hoc power analysis was conducted as a supplementary guide to assess the adequacy of the achieved sample size, rather than as definitive evidence of sufficient power for hypothesis testing. For this purpose, G*Power version 3.1 was used to perform a post hoc power analysis based on a one-group Wilcoxon signed-rank test. The analysis was conducted using a two-tailed test, an alpha level of 0.05, a sample size of 82, and an assumed Cohen’s d of 3.42 (see Supplementary Document for details). The resulting estimated power was 100%, providing additional support for the adequacy of the achieved sample size.

While preparing the survey questionnaire, some questions were selected from a questionnaire conducted by Wolf in 2009 [13]. Given that Wolf’s questionnaire was developed for parents of children with ASD rather than DS, specific items were selected and modified to ensure clarity, relevance, and conceptual appropriateness for the target population and its cultural context. As mentioned by Wolf, the original questionnaire was created by Kreutzer et al. in 1988 to investigate the needs of families with adults suffering from Traumatic Brain Injury (TBI). Waaland et al. then modified it in 1993 to fit the needs of parents of TBI children. The latter was modified by Siklos and Kerns in 2006 [35], tailored to parents of children with ASD and DS, which was modified by Wolf to suit ASD children only [13]. Choosing Wolf’s version (rather than Siklos’ version) is due to the larger pool of items at hand (Wolf has added more items), given the similarity of parents’ needs between the two types of disorders. It should also be noted that our literature search identified other available Family Needs Questionnaires (FNQs). However, these instruments were either developed for families of children with disabilities more generally, such as the Family Needs Survey developed by Bailey and Simeonsson (1988) [8], or were tailored to specific disability populations other than Down syndrome, such as physical disabilities (Alsem et al., 2014) [36] and cerebral palsy (Palisano et al., 2010) [37].

Wolf only verified the content validity quantitatively and did not perform further validation steps. The current questionnaire used Wolf’s items [13] which were deemed suitable to the context of parents with children with DS in Kuwait. Content validity was verified by experts in the field who were fluent in both Arabic and English. They translated Wolf’s questionnaire [13] into Arabic and back-translated it to English to ensure the translation captured the exact meaning of the original version. The translation and back-translation process was conducted by three clinically specialized professionals in the field of Communication Disorders. Two of the professionals held PhD degrees, while the third held a master’s clinical degree. All were fluent in both Arabic and English. Any discrepancies in the translations were primarily resolved through consensus among the translators.

Data collection was conducted from 5 January to 5 April 2020 and was collected from various areas in Kuwait: the questionnaire was distributed to parents in both hard-copy and online formats through Google Forms, with participants given the option to complete it in either Arabic or English.

The questionnaire was developed on the basis of four key factors: educational needs, professional needs, social needs, and primary caregiver support needs. Eight items were considered to measure educational needs, six items were considered to measure professional needs, nine items were considered to measure social needs, and three items were considered to measure primary caregiver support needs. The domains’ formulation stemmed from Wolf’s domain classification [13] of her FNQ, namely: (1) Family, (2) Medical, (3) Educational Needs of Parent, (4) Educational Needs of Child, (5) Social Services, (6) Personal, and (7) Child’s Social Network. Some of the domains were renamed or had their names shortened: the educational needs of parent was amended to “educational needs”; educational needs of the child became “professional needs”; the personal needs were changed to “support” needs; child’s social network became “social needs.” Since we only selected some of Wolf’s FNQ items [13] based on suitability for the Kuwaiti context (based on experts’ judgment at the content validity phase), some of Wolf’s domains [13] were not present in our questionnaire to a considerable extent (namely, the Family and the Medical domains); that is why they were either omitted or merged with other domains (e.g., only one item regarding the child’s health is in our questionnaire; therefore, we attached it to the professional domain).

All four items of need-domains began with the phrase “I need” followed by a statement describing a need related to the child with DS. Responses followed the five-point Likert scale, which were coded from 1 (strongly disagree) to 5 (strongly agree). The participant aggregate score for each domain was his/her average response.

This study was conducted in accordance with the guidelines of the Kuwait University Institutional Review Board (IRB approval code: KU-CLS-20-09-01), the Code of Ethics of the World Medical Association, and the Declaration of Helsinki. Informed consent was obtained prior to survey completion, either electronically through Google Forms or in hard-copy format, depending on the method of survey distribution. To ensure confidentiality, all data were collected anonymously, and no personal identifying information was retained at any stage. Participation was strictly voluntary, and all participants were informed of their right to withdraw at any time without penalty.

IBM SPSS Statistics version 27 was used for the statistical analyses. The software was primarily used to obtain descriptive statistics, including frequency distributions and quartiles. Differences between any two groups in domain scores were assessed using the Mann–Whitney U test, which was preferred to the independent-samples *t*-test because of the clear non-normality of the scores (refer to the Supplementary Document). There was only one instance in which the comparison involved more than two ordinal groups; therefore, the Jonckheere–Terpstra test was used to assess trends in domain scores. Finally, scale reliability was assessed using Cronbach’s alpha.

## 3. Results

### 3.1. Reliability

Cronbach’s alpha coefficients were calculated for the four need domains and the overall scale. Cronbach’s alpha coefficient was evaluated using the guidelines suggested by George & Mallery [38] where >0.9 excellent, >0.8 good, >0.7 acceptable, >0.6 questionable, >0.5 poor, and ≤0.5 unacceptable. As shown in Table 1, Cronbach’s alpha was estimated to be 0.82 for the educational needs domain, 0.79 for the professional needs domain, 0.89 for the social needs domain, 0.83 for the support needs domain, and 0.93 for the overall scale. This roughly suggests good reliability of the four domains (or sub-scales) and excellent reliability for the overall scale.

### 3.2. Sample Description and Demographics

Table 2 shows that the majority of mothers are married (*n* = 70, 85.37%), and the rest are divorced/separated. The table also shows that nearly two-thirds of the mothers (*n =* 54, 65.85%) have 3–5 individuals.

Maternal family members were the most frequent source of support for mothers in caring for their child with DS (*n* = 67, 81.71%), followed by husbands (*n* = 41, 50%). These findings suggest that support for mothers and primary caregivers in this sample was concentrated mainly within close family networks, particularly on the maternal side of the family.

### 3.3. Descriptive Statistics of the Questionnaire Items and Scores per Domain

As shown in Table 3, Table 4, Table 5 and Table 6, all domain items and scores have a median of 5, with IQRs tightly clustered around 5. Part of this result indicates a genuine general need of mothers across all domains (educational, professional, social, and support). It also shows some exaggeration (the elevated scores may also reflect a “wish-list” response tendency, wherein respondents broadly endorse maximum levels of desired support). In other words, the results do not necessarily show a “very high” need across all items, but rather express the existence of a general need of mothers for support across all domains.

### 3.4. The Association Between Mother Characteristic and the Need Domains

Table 7 shows that marital status has a nearly significant association with educational-needs score (*p* = 0.083). Given the small size of the non-married category (*n* = 12, predominantly divorced or separated), such slight deviation from significance is expected due to low statistical power. Assuming a potential underlying effect, we may conclude that those non-married (divorced/separated) have a relatively lower median educational-needs score (median = 4.69, IQR: 4.25–5), as compared to married mothers (median = 5, IQR: 4.72–5). Furthermore, although marital status did not reach statistical significance across the four domains (*p* > 0.10), non-married mothers consistently exhibited lower median scores (across the domains).

Regarding household size, the only domain that demonstrated a significant trend was that of social needs (*p* = 0.012). Statistics provided by the table clearly reflect this as we move towards larger family size (median score = 5 for size within 5 members, 4.83 for family size in the range 6–8, and 3.78 for family size >8).

Receiving support from the husband is not significantly associated with any need domain (all *p*-values > 0.05), while receiving support from a friend or family member is significantly associated with all need domains (*p* < 0.05 in both support types), except that of the professional needs domain (*p* > 0.05 in both support types). By examining the statistics of the significant associations, it can be concluded that the group receiving support has a median score of 5, while the group not receiving support usually has a lower median by 0.12 to 0.67 (and if not, another similar difference may be seen in the 1st quartile of the score). It should be noted that the *p*-values for the non-significant association between receiving support from a friend/family member and the professional needs score are not much larger (*p* = 0.065, in the case of support from a friend, and *p* = 0.077, in the case of support from a family member). One possibility of this slight deviation from significance is the shortage in the sample size of sub-groups (this could have inflated the *p*-value); in other words, there is a possibility of a true association, but it could have been masked by lower sample size. This expectation is supported by the similar pattern observed in the professional needs score and the other domain scores (scores tend to be higher among those receiving support): the median of the professional needs score is 4.83 for the group not receiving support from a family member, while it is 5 for those receiving support from family members. Furthermore, the first quartile for the professional needs score is 4.67 for the group not receiving support from friends, while it is 4.83 for those receiving support from friends (we did not mention the median because it is 5 in both groups).

## 4. Discussion

The descriptive findings showed that all domain items and scores have a median of 5, with IQRs tightly clustered around 5. We have mentioned that part of this result indicates a genuine general need of mothers across all domains (educational, professional, social, and support), while it could also show some exaggeration (the elevated scores may also reflect a “wish-list” response tendency, wherein respondents broadly endorse maximum levels of desired support). Therefore, the results do not necessarily show a “very high” need across all items, but rather express the existence of a general need of mothers for support across all domains. The following paragraphs show literature-based emphasis on the existence of needs across our four domains and the aspects they cover.

### 4.1. Educational Needs Results

Results show that parents have a need for knowledge in DS and behavioral guidance, since being well-educated makes them effective decision-makers for their children. This aligns with recent health literacy data by Akça et al. [39], who found that 63.1% of parents of children with DS possessed inadequate health literacy. On the other hand, there is a strong emphasis on parent-centric education by Çelik et al., Meadan et al., and Stone-Heaberlin et al. [40,41,42], who demonstrated that structured training significantly improves parent–child interactions and parental knowledge implementation. Given such value for information and knowledge, it is important to always communicate news about special programs and services to parents, as indicated by this study’s results. Given the critical value of information and knowledge, establishing effective channels to communicate updates regarding special programs and services remains essential for caregivers, as evidenced by our findings. This directly aligns with Siklos and Kerns [35], who reported that 82% of parents experienced unmet needs concerning service access information.

Furthermore, parents highlighted the need to understand difficult behaviors and to receive validation regarding their decision-making. Feeley and Jones [43] emphasized that providing caregivers with proactive strategies to address early-emerging escape, self-stimulatory, and attention-seeking behaviors is vital.

The psychological burden of decision uncertainty observed in our sample is evident in the qualitative findings of Çelik et al. [40], where parents expressed intense anxiety over whether their home-support actions were correct. This strain is directly associated with lower parental self-efficacy, as addressed by Gilmore and Cuskelly [44].

Finally, the clarity of the future path of the child with DS was one of the needs among mothers in the sample. The distress surrounding future uncertainty in our findings closely mirrors the central qualitative themes reported by Khan et al. and Lee et al. [14,45], who identified long-term future planning among the top unmet caregiver needs. Therefore, it is always important for mothers of children with DS to be surrounded by those who can reassure them about the future and reduce their stress. This can serve as candid outlets where caregivers can express the true emotional challenges. This is even more important so as to remove the feeling of guilt that many mothers may experience when they see their negative feelings regarding their child’s unusual behaviors, as indicated by our results. Connecting parents of a newly diagnosed child with DS to other parents of children with DS can be greatly beneficial, as parents can share their real-life experiences with other parents, which can ultimately assist in parenting a newly diagnosed child with DS. It can also be comforting for parents, as they may become better prepared for an array of situations they may expect and hence develop an enhanced understanding of how to manage hurdles accordingly.

### 4.2. Professional and Clinical Interventions

Within the professional domain, parents expressed a need for collaborative, specialized, and coordinated healthcare and therapeutic services. The demand for concordance among medical professionals regarding best treatment options directly reflects the care fragmentation documented by Fortnum et al. [46], who observed inconsistent clinical pathways and varying treatment philosophies among clinicians managing DS complications.

Mothers, in the samples collected, expressed the need for experienced professionals. Accordingly, Mengoni et al. [47] found that mothers placed great value on Down syndrome-specific knowledge and experience, and they were critical when this was lacking in the professionals they worked with. Mothers, in the samples collected, also expressed a need for licensed and specialized professionals, and this is not strange since the Kuwaiti authors, AlShatti et al. [16] found that the most serious problem encountered by the participants was the unavailability of specialized medical staff to deal with DS and its complications.

The mothers in the samples collected expressed a need for advice/service when the child needs help, which is consistent with what Verstraten-Oudshoorn et al. [48] indicated that parents want to have a care coordinator in constant contact with them. Children’s needs can be emotional too, and their mothers emphasized the need to have counselors or therapists address their child’s emotional needs on a consistent basis. It should be noted that, according to Ben Cheikh et al. [49], the emotional problems were the most reported, and 67.7% of parents asked to be referred to a professional for psychological support. With regard to focusing on mental health and physical health, the mothers expressed the need for regular check-ups for their child. This was emphasized by Turner et al. [50], who also found that poor child health is associated with a higher level of behavior problems and increasing maternal stress over time. It is worth mentioning that the latter author identified the most common health problems (affecting a large percentage of the children): vision and hearing problems and respiratory infections.

### 4.3. Social Inclusion and Family Dynamics

The high priorities observed in the social domain reflect ongoing challenges in community integration, school collaboration, and family social participation. Parents highlighted a need for structured, out-of-school social activities and age-appropriate peer interaction. This matches data from Siklos and Kerns [35], where 72% of the parents reported unmet/partially unmet needs regarding outside-school activities, and 78% reported unmet/partially unmet needs regarding the interaction of the child with others of similar age. The two aforementioned needs are actually connected with the need of mothers in the sample to create outdoor activities, such as outdoor dinners, sometimes per week to fill the gap of few/no friends for some of the children with DS or their usually isolated, sedentary leisure activities—as noted by Oates et al. [51].

In educational settings, the sample showed a need for individualized education plans (IEPs) and robust home-school collaboration. This aligns with Carbone et al. [52], who highlighted (through teacher interviews) the repeated needs of many parents with children with DS to get a customized education for their child or deal with them in a special way. Within the immediate and extended family unit, the mothers indicated the importance of family understanding. AlShatti et al. [16] observed that parents (with DS-children) emphasized the importance of the family’s support, which they believed also included the role of siblings in building the personality of a person with DS. The authors also noted that some caregivers spoke about the need for treating an individual with DS equally in the family so that they may not perceive themselves as being special or different. It was further argued that the child with DS gets saddened when he talks with people who often do not understand him, which could make him become unsocial (this last observation was quoted specifically from a specific mother in the study).

### 4.4. Caregiver Support and Daily Functioning

The elevated scores in the support domain highlight the cumulative physical and operational strain placed on mothers. Needs surrounding personal rest, time with other children, and domestic assistance emerged as important parts of the caregiving needs. The mothers’ need for sleep and rest corresponds with Chawla et al. [53], who documented how sleep difficulties affect their own sleep, daytime function, and family dynamics. The author also noted that parents treat their own sleep loss as an expected burden rather than a health issue.

Furthermore, the mothers’ desire for respite and household support directly intersects with caregiver stress dynamics. Siklos and Kerns [35] noted that 75% of parents experience unmet needs regarding breaks from caregiving responsibilities. Lastly, the mothers’ need for help in housework is empirically reinforced by Campos et al. [54], who established that the absence of daily household help was significantly associated with increased perceived stress (*p* = 0.016) among parents of individuals with DS.

### 4.5. Are Findings Consistent Across Different Demographic Groups of Mothers?

Unfortunately, this was not tested with several demographic variables (tests were only conducted with regard to marital status, family size and type of relationship with the supporter, if any), which has will be addressed in Section 7.

With reference to the marital status, data showed nonsignificant association with need-domains, however descriptive statistics show a consistently and relatively higher median scores among married women, as compared to others (divorced/separated). This descriptive pattern may warrant further investigation, particularly to examine whether it is related to perceived unequal distribution of the caregiving burden among some mothers. The role of burden distribution in this regard has been addressed by Roach et al. [55].

With regard to family size, the only clear and significant association was the one with social needs, and this is actually logical, since more siblings could fill some of the social needs gaps, which was emphasized by AlShatti et al. (2021) [16].

Finally, with regard to the type of relationship of other supporters with mothers, it was found that having a friend or family support system was significantly associated with almost all need domains (they have only a nearly significant association with the professional needs, which could be due to a shortage in the sample). Statistics show that the need was higher on the side of those getting support. However, from the research conducted, it became evident that association does not directly imply any causation; it could possibly refer to the fact that those with high needs across all domains would naturally need more support from others such as family members or friends.

### 4.6. Further Discussion

This pattern of having at least a considerable need across all domains reinforces the study’s central argument that post-diagnostic support cannot be addressed through single-domain interventions, and is consistent with Hart and Neil [9], who similarly found that caregivers of children with DS report gaps across multiple support domains rather than in isolated areas.

The social support source from husband, family, friends, etc., further contextualizes these findings within the Kuwaiti family structure. Maternal family members were the predominant source of support for caregivers (*n* = 67, 81.71%), while community and peer-based sources were rarely cited. This pattern reflects the collectivist and family-centered nature of Kuwaiti society [56], where caregiving responsibilities and support networks are channeled primarily through extended family rather than formal community structures [6]. Significantly, this has direct implications for intervention design. Support programs modeled on Western peer-network or community-based approaches may have limited reach in this context [57]. Interventions in Kuwait may prove to be more effective if they are designed to work within and strengthen existing extended family networks, particularly by equipping maternal family members with accurate information about DS and practical caregiving guidance.

Findings of existing professional needs among mothers (e.g., looking for a trained counselor or therapist and for special programs and services) are important because they suggest that professional support after diagnosis is not limited to the communication of information alone but can also include supporting primary caregivers in understanding the types of developmental services their child may need over time. This is particularly relevant because previous research has shown that families of individuals with DS often report unmet professional support needs and low satisfaction with speech and language therapy services, highlighting the importance of clearer guidance and referral pathways after diagnosis [58].

## 5. Clinical Implications

The findings of this study provide an empirical basis for a multidimensional approach to support mothers of children with DS. High ratings were observed across all domains of need, with median scores of 5 or close to 5, indicating substantial needs across different aspects of support. These findings suggest that policymakers within the health sector in Kuwait may need to consider coordinated support across the professional, social, educational, and practical support dimensions. Such an approach could include parent-focused workshops and training within health centers, as well as recreational/leisure activities that provide opportunities for parents to meet and connect with one another, hence addressing both educational and social needs. The findings may also provide implications for the development of area-based centers dedicated to children with DS and other developmental conditions, which could allow more focused support for children and families while reducing the competing demands of multipurpose hospitals and health centers and potentially creating greater opportunities for specialized professionals. Even though access to specific professional services was not directly assessed in the present study, such centers may also facilitate access to specialized services such as speech-language pathology, which has been identified in previous research as an important component of early intervention and communication support for children with DS [20,21].

## 6. Conclusions

The findings of this study suggest that the support needs of mothers of children with DS in Kuwait following postnatal diagnosis are not limited to one isolated area. Therefore, the results indicate that support following postnatal diagnosis should not be limited to the communication of the diagnosis itself, but should also include emotional support, access to trained professionals, social support, and practical caregiver support. These findings are consistent with another published article on caregivers’ needs which found, through a mixed-method study, that caregivers of children with DS require support across multiple domains and experience gaps in accessible social and community support. In addition, there was clearly a need for practitioners to be sympathetic, well-informed and good communicators [9]. Such support can help caregivers feel more informed, supported, and better able to respond to the needs of their child.

The high response rate from mothers compared to the limited participation of fathers may reflect the prominent role of mothers in caregiving within the Kuwaiti family support structure [16,59]. Findings from a recent study conducted in Kuwait involving parents of children with ASD also align with this interpretation, as mothers were more frequently identified as primary caregivers than fathers [60]. Although this viewpoint is gradually changing [59], the observed participation pattern reinforces the need for interventions that specifically target maternal mental health and professional guidance [16].

Overall, this study contributes to a growing body of evidence that post-diagnostic support for caregivers of children with DS must be understood and delivered as a coordinated, multidimensional process rather than a single clinical encounter. In the Kuwaiti context, where this area has been underexamined, these findings offer an empirical foundation for the development of more integrated and culturally responsive support pathways that address caregivers’ educational, professional, social, and practical needs holistically.

## 7. Limitations and Future Studies

The present study did not receive enough responses from fathers (*n* = 4, before we excluded them), despite their equally key role in child development. Although this is culturally representative, future studies should consider conducting surveys targeting the male populations of children with DS to enable drawing a scientific analysis of parent dynamics rather than only that of the women as primary caregivers. This will most likely reflect the cultural reality of the region in which mothers typically serve as the primary home caregivers and the main point of contact for developmental and medical services for their children. However, as this viewpoint is evolving, fathers are becoming more proactive in their children’s development and well-being. Future surveys targeting fathers would not only provide us with a balanced perspective, but could also significantly increase our sample size, which, in turn, can provide us with the opportunity to draw more meaningful conclusions. As such, further validation in larger caregiver samples would strengthen confidence in the measures.

The study did not collect data on several demographic variables; it was limited to gender, marital status, family size, and the nature of the relationship with individuals providing support. Following the exclusion of fathers from the analysis, gender was no longer applicable as a variable for comparison. Therefore, future studies should collect a broader range of demographic characteristics and ensure sufficiently large sample sizes to allow comparisons across demographic groups in terms of their support needs. This would help determine whether specific groups differ from the overall pattern of needs.

Since all measures were self-reported and collected at one point in time, the possibility of common-method bias and social desirability effects is high. Moreover, while Kuwait ranks amongst the highest in digital literacy, the distribution method using Google Forms might have also excluded caregivers with lower digital literacy or those not connected to support networks directly associated with our convenience sampling.

Future studies that target pediatricians’ own perspectives in Kuwait Healthcare settings are another area worth surveying to receive a fuller perspective of DS diagnosis communication and disclosure practices.

The questionnaire items were selected from a previous study (Wolf, 2009) [13] and distributed amongst four domains without a full procedure for validation. The validation was restricted to content validity (expert review, with translation and back-translation); it lacked face and construct validity (the latter is usually done with EFA/CFA).

The high ceiling of the scores could be attributed to two factors: a genuine feeling of need among mothers, and questionnaire wording and “wish-list” response tendency (tendency of mothers to get the maximum benefits and support). Therefore, while results show a general feeling of needs, it is difficult to know which needs are more important or have a higher priority (presented in the Supplementary Materials). A strong form of data collection design would be one that measures the degree of importance of certain needs, followed by measuring whether they are met or not (in degrees).

## Figures and Tables

**Table 1:** Reliability table.

Scale	No. of Items	α
**Educational needs (Factor 1)**	8	0.82
**Professional needs (Factor 2)**	6	0.79
**Social needs (Factor 3)**	9	0.89
**Primary caregiver support needs (Factor 4)**	3	0.83
**Overall Score**	**26**	**0.93**

**Table 2:** Characteristics of participants.

Variable	Categories	Frequency (Total = 82)	%
**Marital status**	Married	70	85.37
	Divorced	5	6.10
	Separated	5	6.10
	Widow	2	2.44
**Household size**	<3	15	18.29
	3–5	54	65.85
	6–8	10	12.20
	>8	3	3.66
**Individuals providing support to the mother**	Husband	41	50.00
	Friend	28	34.15
	Family member	67	81.71
	Family friend	2	2.44
	Other	20	24.39

**Table 3:** Median (IQR) for responses to the items concerning the educational needs.

Item	Median (IQR)
I need to be well-educated about my child’s Down syndrome in order to be an effective decision maker regarding the needs of my child.	5 (5–5)
I need to be educated as to why my child acts in ways that are different, difficult or unusual.	5 (5–5)
I need to be shown that my opinions are used in planning my child’s treatment, therapies, or education	5 (4–5)
I need to be educated on what to do when my child is acting unusually or is displaying difficult behaviors.	5 (4–5)
I need to understand if I am making good decisions about my child.	5 (5–5)
I need for myself to be reassured that it is not uncommon to have negative feelings about my child’s unusual behaviors.	5 (5–5)
I need for myself to help deal with my fears about my child’s future.	5 (5–5)
I need to obtain information about special programs and services available to my child for my own education.	5 (5–5)
**Overall Score**	**4.94 (4.59–5)**

**Table 4:** Median (IQR) for responses to the items concerning the professional needs.

Item	Median (IQR)
I need different professionals such as physicians and medical doctors to agree on the best treatment options for my child.	5 (5–5)
I need to have a professional to turn to for advice or services when my child needs help	5 (5–5)
I need my child’s counselor or therapist to have experience working with children with the same disorder as my child.	5 (5–5)
I need to have counselors or therapists address my child’s emotional needs on a consistent basis.	5 (5–5)
I need my child to have social skills training by a licensed professional, therapist, or counselor.	5 (5–5)
I need medical screening tests for my child every so often.	5 (5–5)
**Overall Score**	**5 (4.67–5)**

**Table 5:** Median (IQR) for responses to the items concerning the social needs.

Item	Median (IQR)
I need weekend and after-school social activities for my child.	5 (5–5)
I need home-school collaboration for my child.	5 (5–5)
I need for my child to interact with others his/her own age.	5 (5–5)
I need my child’s school to set up an individualized education plan for my child.	5 (5–5)
I need my other children to have counseling.	5 (5–5)
I need to have other family members understand my child’s problems.	5 (5–5)
I need for my family to go out for dinner together a certain number of times each week.	5 (5–5)
I need for other children to feel comfortable around my child when in social settings.	5 (5–5)
I need to have a range of social services available for my child.	5 (5–5)
**Overall Score**	**5 (4.78–5)**

**Table 6:** Median (IQR) for responses to the items concerning the support needs.

Item	Median (IQR)
I need for myself to have time to spend alone with my other children in my family.	5 (5–5)
I need for myself to get enough rest or sleep.	5 (5–5)
I need for myself to have help with housework.	5 (5–5)
**Overall Score**	**5 (4.67–5)**

**Table 7:** The association between mother characteristics and the need domains.

Characteristic Variable	Categories	N	Median (IQR) for:			
			Educational Needs	Prof. Needs	Social Needs	Support Needs
**Marital status**	Married	70	5 (4.72–5)	5 (4.67–5)	5 (4.78–5)	5 (4.67–5)
	Other	12	4.69 (4.25–5)	4.92 (4.5–5)	4.94 (4.69–5)	4.83 (4.08–5)
	***p*** **-Value**		**0.083**	**0.467**	**0.577**	**0.122**
**Household size**	<3	15	5 (4.88–5)	5 (4.83–5)	5 (4.89–5)	5 (4.67–5)
	3–5	54	4.88 (4.59–5)	5 (4.67–5)	5 (4.78–5)	5 (4.92–5)
	6–8	10	5 (4.84–5)	5 (4.29–5)	4.83 (4.44–5)	5 (4.33–5)
	>8	3	4 (3.5– *)	4.67 (3.33– *)	3.78 (2.67– *)	3 (3– *)
	***p*** **-Value**		**0.526**	**0.193**	**0.012**	**0.125**
**Husband**	No	41	4.88 (4.5–5)	5 (4.5–5)	5 (4.67–5)	5 (4.67–5)
	Yes	41	5 (4.75–5)	5 (4.83–5)	5 (4.89–5)	5 (4.67–5)
	***p*** **-Value**		**0.147**	**0.329**	**0.249**	**0.487**
**Friend**	No	54	4.88 (4.5–5)	5 (4.67–5)	4.89 (4.64–5)	5 (4.33–5)
	Yes	28	5 (4.88–5)	5 (4.83–5)	5 (5–5)	5 (5–5)
	***p*** **-Value**		**0.033**	**0.065**	**0.002**	**0.014**
**Family member**	No	15	4.75 (4–5)	4.83 (4.33–5)	4.67 (4.11–4.89)	4.33 (3.33–5)
	Yes	67	5 (4.75–5)	5 (4.67–5)	5 (4.89–5)	5 (5–5)
	***p*** **-Value**		**0.046**	**0.077**	**<0.001**	**<0.001**

* Not calculated due to shortage in sub-sample size (*n* = 3).

## Data Availability

Due to privacy, data supporting the findings of this investigation are available from the corresponding author upon request.

## Supplementary Materials

The following supporting information can be downloaded at: https://www.mdpi.com/article/10.3390/healthcare14203368/s1: Supplementary Figure S1: Frequency Distribution of Educational Needs Score (Histogram). Supplementary Figure S2: Frequency Distribution of Professional Needs Score (Histogram). Supplementary Figure S3: Frequency Distribution of Social Needs Score (Histogram). Supplementary Figure S4: Frequency Distribution of Support Needs Score (Histogram). Supplementary Table S1: Frequency of Min/Max Possible Scores. Supplementary Table S2: Mean, SD and Cohen’s d for each Domain Score. Supplementary Table S3: Wolf’s QNS Items and their Corresponding Modified Version.
