# Supplementary material for: Support Needs of Mothers of Children with Down Syndrome in Kuwait
Source: Healthcare (Basel). 2026 Oct 9;14(20):3368. doi: 10.3390/healthcare14203368 (PMC13704526; doi:10.3390/healthcare14203368)
Supplement: Supplementary file 1 [file healthcare-14-03368-s001.zip › healthcare-4507629-supplementary.pdf]

Support Needs of Mothers and Primary Caregivers of Children with Down Syndrome in Kuwait
Supplementary Tables and Figures

Supplementary Figures

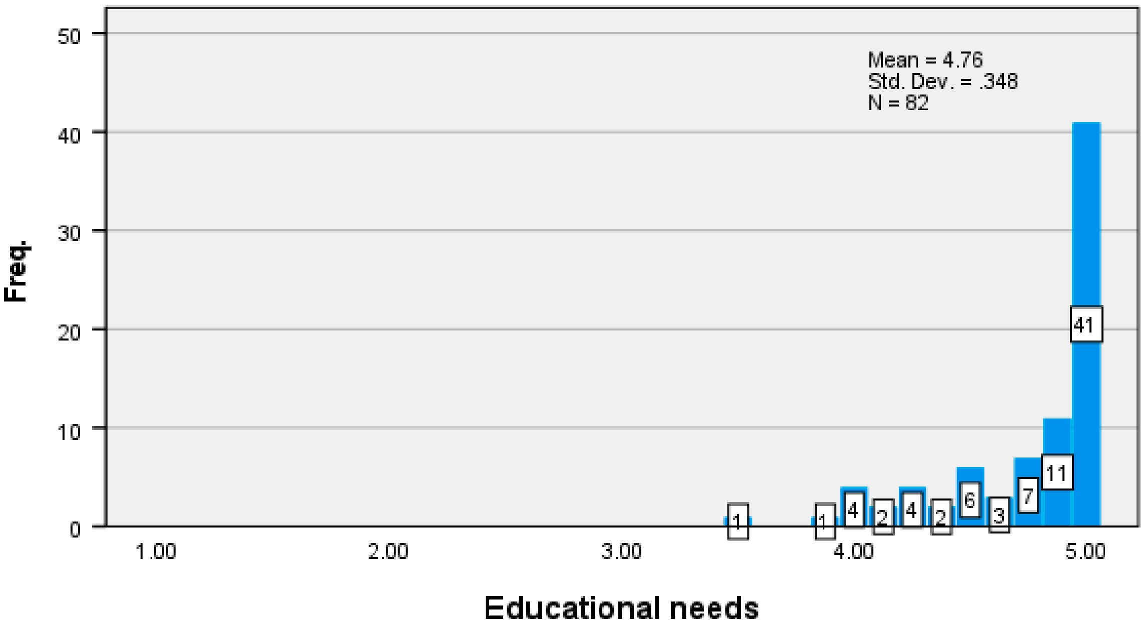

Supplementary Figure S1: *Frequency Distribution of Educational Needs Score (Histogram)*

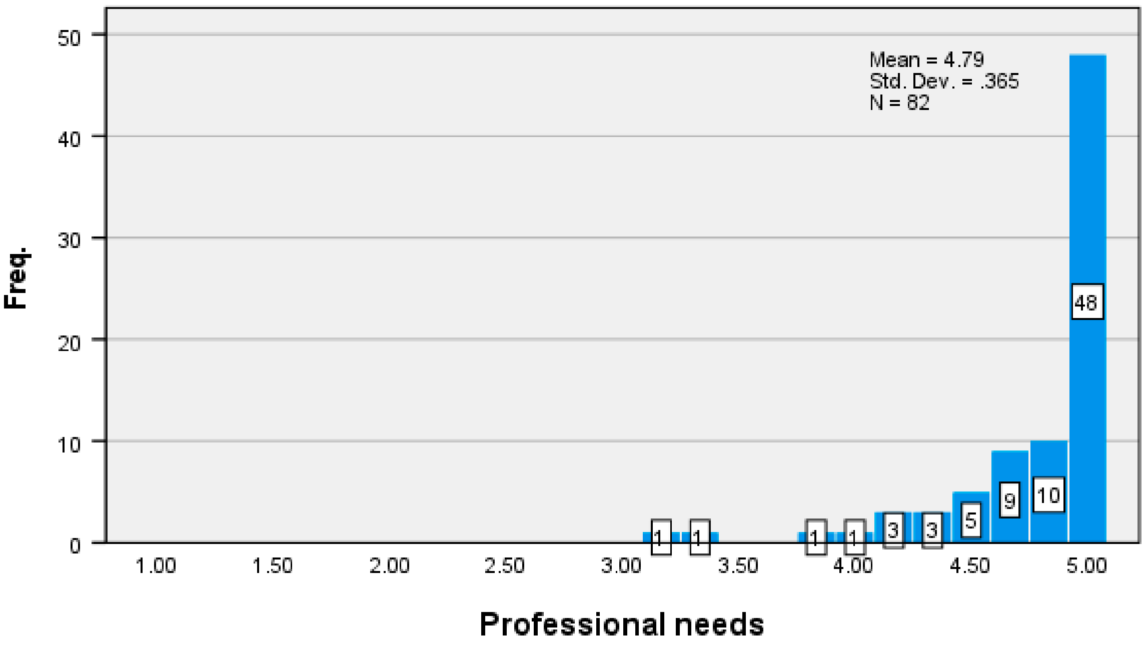

Supplementary Figure S2: *Frequency Distribution of Professional Needs Score (Histogram)*

Support Needs of Mothers and Primary Caregivers of Children with Down Syndrome in Kuwait
Supplementary Tables and Figures

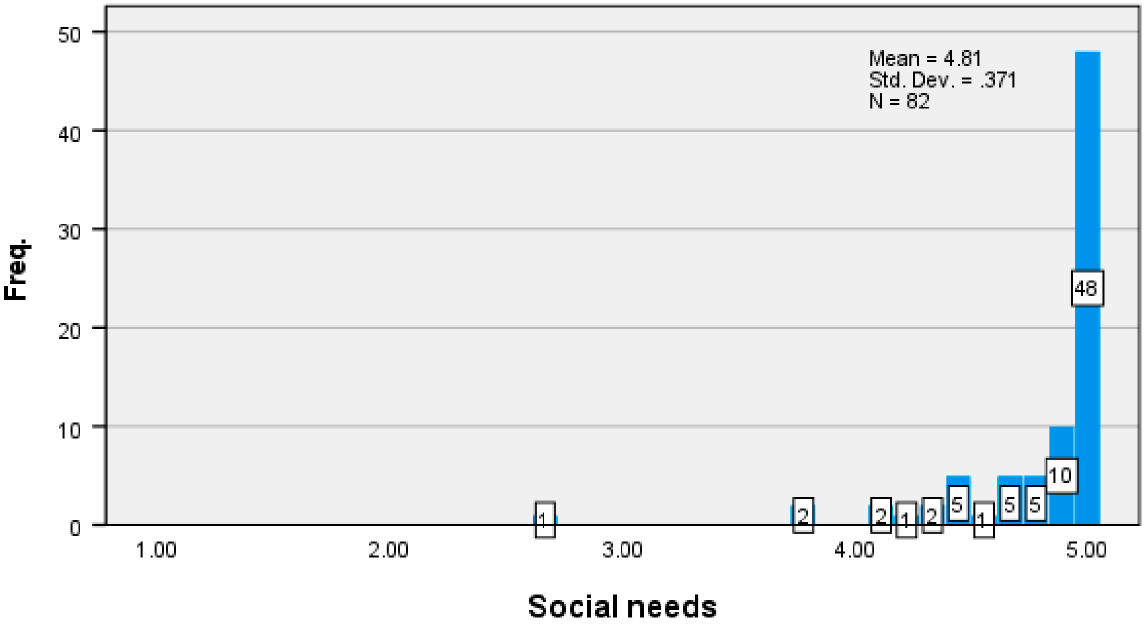

Supplementary Figure S3: *Frequency Distribution of Social Needs Score (Histogram)*

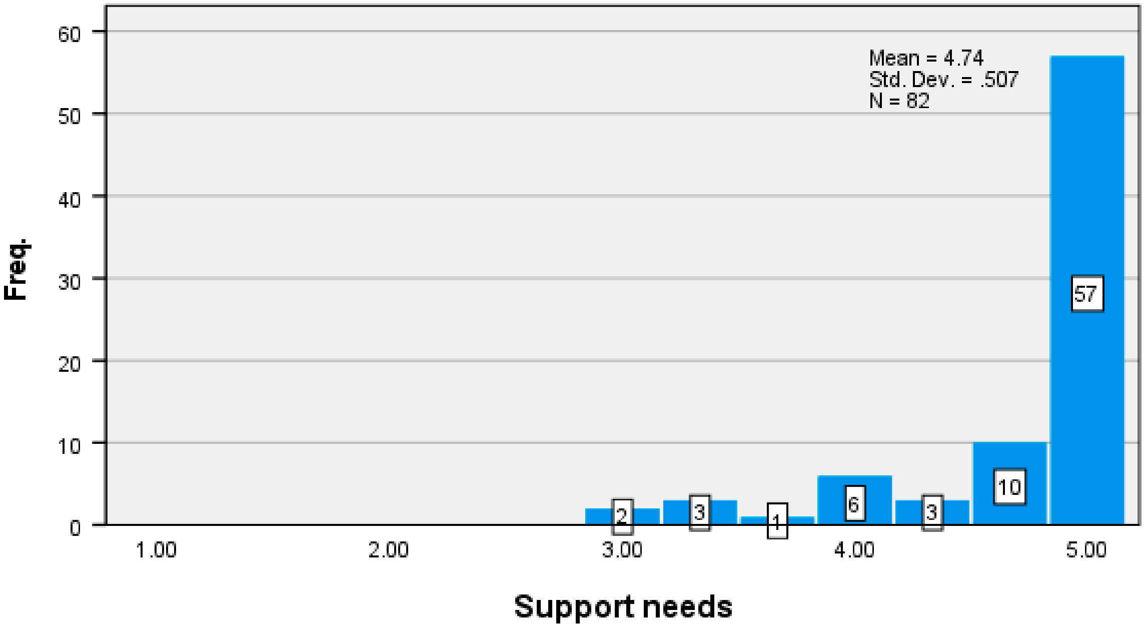

Supplementary Figure S4: *Frequency Distribution of Support Needs Score (Histogram)*

Support Needs of Mothers and Primary Caregivers of Children with Down Syndrome in Kuwait
Supplementary Tables and Figures

Supplementary Tables

Supplementary Table S1: *Frequency of Min / Max Possible Scores*

Score Category	Educational Needs		Professional Needs		Social Needs		Support Needs	
	N	%	N	%	N	%	N	%
**Minimum possible score (0)**	0	0.00	0	0.00	0	0.00	0	0.00
**Scores < 4.50**	14	17.07	10	12.20	13	15.85	15	18.29
**Scores near the maximum (4.50 to 4.99)**	27	32.93	24	29.27	21	25.61	10	12.20
**Maximum possible score (5)**	41	50.00	48	58.54	48	58.54	57	69.51

Supplementary Table S2: *Mean, SD and Cohen's d for each Domain Score*

Domain	Overall Score Statistics		
	**Mean**	**SD**	**Cohen's d**
**Educational needs**	4.76	0.35	5.06
**Professional needs**	4.79	0.36	4.90
**Social needs**	4.81	0.37	4.87
**Support needs**	4.74	0.51	3.42

Note: Cohen's dd was calculated solely for the purpose of conducting the post-hoc power analysis; it did not correspond to a hypothesis tested in the present study. The reference mean was set at 3.0, corresponding to the midpoint of the five-point Likert scale. For the power analysis, the lowest observed Cohen's dd among the four domains was used as a conservative assumption, representing the least favorable scenario for statistical power.

Supplementary Table S3: *Wolf's QNS Items and their Corresponding Modified Version*

Wolf's QNS Item	Modified Version of the Item
I need to be well-educated about my child's autism (autism spectrum disorder) in order to be an effective decision maker regarding the needs of my child.	I need to be well-educated about my child's down syndrome in order to be an effective decision maker regarding the needs of my child.
I need social services continuously rather than only in times of crisis.	Not selected
I need to have consistent physical therapy for my child.	Not selected
I need for my child to have friends of his/her own.	Not selected
I need different professionals such as psychologists/counselors to agree on the best therapy options for my child.	Not selected

I need different professionals such as physicians and medical doctors to agree on the best treatment options for my child.	I need different professionals such as physicians and medical doctors to agree on the best treatment options for my child.
I need to have a therapist/counselor to turn to for advice or therapy services when my child needs help.	I need to have a professional to turn to for advice or services when my child needs help
I need to have a physician/medical doctor to turn to for advice when my child needs help.	Not selected
I need for my child to have consistent behavioral therapy.	Not selected
I need support from other family members in taking care of my child.	Not selected
I need weekend and after-school social activities for my child.	I need weekend and after-school social activities for my child.
I need therapists or counselors who have expertise working with children who have autism (autism spectrum disorder) like my child.	Not selected
I need to have consistent occupational therapy for my child.	Not selected
I need to be educated as to why my child acts in ways that are different, difficult or unusual.	I need to be educated as to why my child acts in ways that are different, difficult or unusual.
I need for myself to have time to spend alone with my other children in my family.	I need for myself to have time to spend alone with my other children in my family.
I need to be educated on what to do when my child is acting unusually or is displaying difficult behaviors.	I need to be educated on what to do when my child is acting unusually or is displaying difficult behaviors.
I need to obtain information and knowledge regarding my child's therapeutic or educational progress.	Not selected
I need to be educated on how much to let my child do by himself/herself.	Not selected
I need for my child to engage in social activities with others his/her own age.	Not selected
I need to have my child's school set up consistent speech therapy for my child as part of his/her education plan.	Not selected
I need for myself to have help with housework.	I need for myself to have help with housework.
I need for myself to get enough rest or sleep.	I need for myself to get enough rest or sleep.
I need for myself to get a break from my responsibilities.	Not selected
I need for myself to spend time with my friends.	Not selected
I need to understand if I am making good decisions about my child.	I need to understand if I am making good decisions about my child.

I need for medical professionals working with my child to understand the health needs of my child.	Not selected
I need for counselors or therapists working with my child to understand the emotional needs of my child.	Not selected
I need for other children to feel comfortable around my child when in social settings.	I need for other children to feel comfortable around my child when in social settings.
I need to have other family members understand my child's problems.	I need to have other family members understand my child's problems.
I need to have my child's after-school friends to understand his/her problems.	Not selected
I need for myself to express feelings about my child with a parent who has a child with autism (autism spectrum disorder).	Not selected
I need my child's social services to continue throughout the summer months and school breaks.	Not selected
I need for myself to be reassured that it is not uncommon to have negative feelings about my child's unusual behaviors.	I need for myself to be reassured that it is not uncommon to have negative feelings about my child's unusual behaviors.
I need for myself to help deal with my fears about my child's future.	I need for myself to help deal with my fears about my child's future.
I need for myself to have help in remaining hopeful about my child's future.	Not selected
I need for myself to be encouraged to ask for help from others.	Not selected
I need my other children to have counseling.	I need my other children to have counseling.
I need to obtain information about special programs and services available to my child for my own education.	I need to obtain information about special programs and services available to my child for my own education.
I need to be personally shown respect by those working with my child.	Not selected
I need to have my child's teacher to be able to teach other students in the classroom about his/her unusual behaviors in a constructive way.	Not selected
I need for myself to have time to spend alone with my partner.	Not selected
I need my child's school to set up an individualized education plan for my child.	I need my child's school to set up an individualized education plan for my child.
I need my child to have a teacher's aide with him/her at school who has knowledge about, or experience with, working with children with the same disorder as my child.	Not selected

I need to have medical professionals address my child's medical needs on a consistent basis.	Not selected
I need to have counselors or therapists address my child's emotional needs on a consistent basis.	I need to have counselors or therapists address my child's emotional needs on a consistent basis.
I need for my family to agree on decisions regarding our developmentally delayed child.	Not selected
I need my child's physician or medical doctor to have experience working with children with autism (autism spectrum disorders).	Not selected
I need my child's counselor or therapist to have experience working with children with the same disorder as my child.	I need my child's counselor or therapist to have experience working with children with the same disorder as my child.
I need for my family to go out for dinner together a certain number of times each week.	I need for my family to go out for dinner together a certain number of times each week.
I need for my family to take more vacations each year.	Not selected
I need to take week-long vacations by myself each year.	Not selected
I need to have a range of medical services available for my child.	Not selected
I need to have a range of social services available for my child.	I need to have a range of social services available for my child.
I need hospital services that are readily available for my child.	Not selected
I need medical screening tests for my child every so often.	I need medical screening tests for my child every so often.
I need a pediatrician who is able to help my child meet his/her nutritional and physical needs.	Not selected
I need medical professionals to agree on the best ways to treat my child.	Not selected
I need to be educated regarding my child's therapeutic and educational progress.	Not selected
I need my child's school environment to have more structure or routine.	Not selected
I need my child to have access to visual aid teaching while at school.	Not selected
I need home-school collaboration for my child.	I need home-school collaboration for my child.
I need my child to be in group therapy for his/her behavioral issues.	Not selected
I need my child to have some type of sensory integration therapy.	Not selected
I need my child to have social skills training by a licensed professional, therapist, or counselor.	I need my child to have social skills training by a licensed professional, therapist, or counselor.

I need for my child to interact with others his/her own age.	I need for my child to interact with others his/her own age.
I need to have more structure at home with my family.	Not selected
I need for my child to desire social interactions with other children.	Not selected
N/A	I need to be shown that my opinions are used in planning my child's treatment, therapies, or education*
